# Modelling habitat suitability for *Moringa oleifera* and *Moringa stenopetala* under current and future climate change scenarios

**DOI:** 10.1038/s41598-023-47535-5

**Published:** 2023-11-18

**Authors:** Jintu Kumar Bania, Jyotish Ranjan Deka, Animekh Hazarika, Ashesh Kumar Das, Arun Jyoti Nath, Gudeta W. Sileshi

**Affiliations:** 1 Department of Ecology and Environmental Science, Assam University, Silchar, Assam India; 2 Wildlife Institute of India, Dehradun, Uttarakhand 248001 India; 3 Department of Plant Biology and Biodiversity Management, Addis Ababa, Ethiopia

**Keywords:** Plant domestication, Computational biology and bioinformatics, Ecology

## Abstract

*Moringa oleifera* Lam and *Moringa stenopetala* (Baker f.) Cufod are being widely promoted as multipurpose trees across the tropics for their nutritional, medicinal and soil health benefits. Different parts of these species are edible, have therapeutic values and their seeds are used for water purification. Although the two species are similar in many ways, they have contrasting distributions. However, their current promotion is not guided by adequate knowledge of the suitability of the target areas. Information is also scanty on the suitability of habitats for these species under the current and future climate change scenarios. Therefore, the objective of this study was to predict the habitat suitability of *M. oleifera* and *M. stenopetala* under current and future climate change scenarios using an ensemble of models assuming four shared socio-economic pathways, namely, SSP1-2.6, SSP2-4.5, SSP3-7.0, and SSP5-8.5 for 2050 and 2070. The results suggest that areas that are highly suitable for *M. oleifera* will increase by 0.1% and 3.2% under SSP1-2.6 to SSP5-8.5 by 2050, respectively. By 2070, the area suitable for *M. oleifera* would likely decrease by 5.4 and 10.6% under SSP1-2.6 and SSP5-8.5 scenarios, respectively. The habitat that is highly suitable for *M. stenopetala* was predicted to increase by 85–98% under SSP3-7.0 and SSP5-8.5 scenarios by 2050 and by 2070, while suitable areas could increase by up to 143.6% under SSP5-8.5. The most influential bioclimatic variables for both species were mean diurnal temperature range, mean temperature of driest quarter, precipitation of wettest month, and isothermality. Additionally, soil pH, elevation and water holding capacity were influential variables in the distribution of *M. oleifera*, while soil pH, soil salinity and slope were influential in *M. stenopetala* distribution. This study has provided baseline information on the current distribution and possible future habitat suitability, which will be helpful to guide formulation of good policies and practices for promoting *Moringa* species outside their current range.

## Introduction

*Moringa oleifera* Lam. and *Moringa stenopetala* (Baker f.) Cufod. are among the 13 or more known species in the monogeneric family Moringaceae^1^. Although the two species are similar in many ways, they have contrasting distributions, which makes them interesting both from a theoretical and practical perspective. How some species have wide distribution, while others are restricted in their distribution has fascinated biogeographers and ecologists for years. While *M. oleifera* is native to the Indian subcontinent, it is extensively cultivated in countries like the Philippines, Cambodia, and the Caribbean Islands^2^. On the other hand, the native range of *M*. *stenopetala* is restricted to a small part of East Africa including Ethiopia and northern Kenya^3–5^. Indeed, *M*. *stenopetala* is only known in the wild from northern Kenya, but it is widely cultivated in Ethiopia^4^. Reports from Djibouti, Malawi, Senegal, Somalia, Sudan and Uganda are probably based on recent introductions^4^.

Both species are promoted in the tropics as multipurpose species in agroforestry systems to provide animal fodder, human food and climate change mitigation^6,7^. For example, the Food and Agriculture Organization (FAO) has been promoting *Moringa* in agroforestry programs since the 1990s^8^. *Moringa* trees have several uses in different parts of the globe. Almost every part of the tree is edible and highly nutritious^9^ and contain many essential minerals and vitamins^10,11^. *Moringa oleifera* is known to be rich in proteins, vitamin A, minerals, essential amino acids, antioxidants, and flavonoids, as well as isothiocyanates. The extracts also have multiple nutraceutical or pharmacological functions including anti-inflammatory, antioxidant, anti-cancer, hepatoprotective, neuroprotective, hypoglycemic, and blood lipid-reducing functions^12^. Furthermore, the *Moringa* tree has a significant contribution to traditional medicines in Asia and Africa. Different parts of *M. oleifera* and *M. stenopetala* have been used in traditional medicine to treat several health issues such as ascites, rheumatism and snake bites, and cardiac and circulatory stimulants^4,7,13^. The leaves of *M. oleifera* can also be used as natural plant growth promoter as they contain several growth hormones and mineral elements^14^.

*Moringa* is well adapted to adverse conditions where other plants have a very low level of survival rate^15^. Recently, intercropping of *Moringa* with other crops has been promoted because it improves yields, providing food and cash^16^. In some regions, *Moringa* seeds are used for water purification, and this is gaining interest among researchers as chemical water treatment is costly especially in many developing nations^17^. The use of *Moringa* seed was shown to reduce the turbidity of water by up to 90% and microbial growth by 95%, and hence a cost-efficient solution for water pollution^18^. It was reported that the *Moringa* plant is more effective in removing water turbidity than other natural coagulants^19^. In addition, *Moringa* products also have high commercial value in many countries, and their cultivation can help farmers to generate income^7,20,21^.

The cultivation of *M. oleifera* and *M. stenopetala* is also increasingly recommended as a climate-smart solution^16,22^. As a result, planting of *Moringa* in agroforestry systems has increased^22^. In countries like Niger, Ethiopia and Indonesia, different *Moringa*-based agroforestry systems are being practised. In Ethiopia, root crops such as *Ensete ventricosum*, *Ipomoea batatas*, *Colocasia esculenta*, *Manihot esculenta* and *Dioscorea alata* are intercropped with *Moringa oleifera*^23^. Similarly, in Niger cereal crops, fruiting trees, henna, and lettuce are grown in *Moringa-*based agroforestry systems^24^. Food crops like peanuts, corn and cassava are being cultivated with *Moringa oleifera* tree in alley cropping system in Indonesia^25^. In India, several efforts are being made to popularise *Moringa-*based agroforestry systems^22^. Rathore et al*.*^26^ showed that *Moringa-*mung bean-potato could be one of the most productive agroforestry systems, which could produce goods up to 36.2 Mg ha^−1^.

While *Moringa* species have been hailed for their multipurpose uses, concerns have also been raised about their potential to become invasive alien species with their increasing commercialisation. For example, in South Africa, *Moringa* is on the Species Under Surveillance for Possible Eradication or Containment Targets, where it is classified as Category E (i.e., fully invasive)^7^.

Currently, the two species are being promoted outside their known geographic range without adequate knowledge of suitability of the target areas. Information is lacking on the current distribution of *Moringa* species and the future habitat suitability under climate change. In this study, we applied an ensemble of species distribution models (SDMs) to map potentially suitable habitats with the aim to inform conservation and promotion of the species. SDMs are an increasingly important tool in ecology, biogeography and conservation science^27^. SDMs are able to predict areas where environmental conditions are appropriate for the survival of a species, even where it is not currently present, which is called the potential distribution or fundamental niche^28^. SDMs are useful in quantifying the correlation between environmental factors and the distribution of species^29,30^. The use of ensemble models is also increasing in SDMs because of the opportunity they provide for evaluation of possible climate change impacts on plant species and identifying populations that are threatened and areas where urgent conservation measures are needed^28^. To predict an outcome, ensemble modelling creates multiple models. These models can use different algorithms (regression/machine learning) or datasets for training. The ensemble model combines the predictions of each base model into one final prediction for new data. The goal of ensemble modelling is to lower the prediction error^31,32^. There are not many studies on ensemble modelling of the distribution of *Moringa* species; the only one is on the mapping of *Moringa oleifera* in South Africa^33^. Hence, we assessed the predicted habitat suitability of these *Moringa* species in consideration of different environmental variables. Similarly, it is also hypothesized that both the *Moringa* species are expected to change their habitat ranges with the changing of environmental conditions. Therefore, this study aims to estimate the current and future habitat suitability of *M. oleifera* and *M. stenopetala* in the tropical regions under climate change scenarios and to identify the influential environmental factors affecting the spatio-temporal distribution of *Moringa* species. The results of this exercise are expected to inform development of good policies and practices for promoting these species and in the event that they become invasive^34^.

## Results

### Model performance

With an AUC of ≥ 0.85, the ensemble models used here demonstrated a moderate performance. The various performance metrics used for comparing the individual models and ensemble model are summarized in Table 1. In the case of *M. stenopetala*, RF, MaxEnt and BRT predicted the current suitability with an AUC of ≥ 0.93. Similarly, the TSS of the model indicated good predictive performance (TSS value ≥ 0.55) for the distribution of *M. oleifera* except for CART (TSS < 0.51). The TSS values for *M. stenopetala* indicated a very good performance (> 0.70) for all models except CART. Therefore, we excluded the CART model from the ensemble function for both species due to its lower accuracy. In *M. oleifera*, RF outperformed all other models, with a high COR (0.67) and a comparatively lower deviance (0.8). In the case of *M. stenopetala*, RF performed better (COR = 0.78; deviance = 0.12) than all other models.

**Table 1:** Performance evaluation of SDMs using different statistical parameters for the current distribution of *Moringa oleifera* and *Moringa stenopetala* in Tropical countries.

Methods	*M. oleifera*				*M. stenopetala*			
	AUC	COR	TSS	DEVIANCE	AUC	COR	TSS	DEVIANCE
RF	0.89	0.67	0.66	0.80	0.95	0.78	0.85	0.12
MaxEnt	0.85	0.59	0.61	0.92	0.96	0.74	0.89	0.5
MARS	0.85	0.58	0.58	0.93	0.87	0.66	0.78	0.36
BRT	0.85	0.58	0.59	1.03	0.93	0.72	0.80	0.16
CART	0.79	0.49	0.51	1.14	0.79	0.64	0.66	0.23
SVM	0.85	0.60	0.63	0.91	0.89	0.75	0.81	0.14
Overall	0.85	0.59	0.60	0.96	0.90	0.72	0.80	0.25

### Current habitat suitability

**Figure 1:**
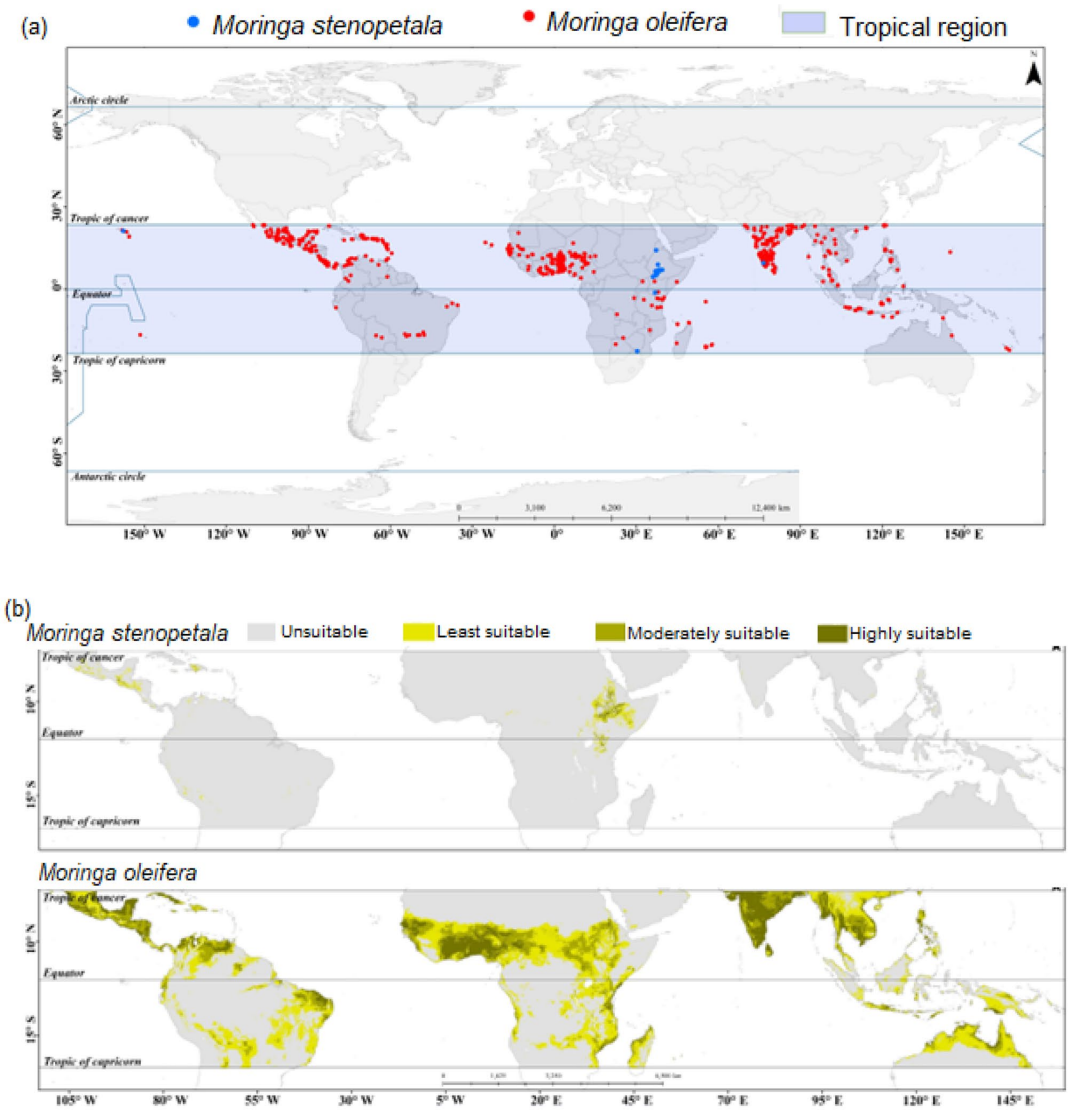
Map of locations of data used for the species distribution modelling (**a**) and the current distribution of *Moringa oleifera* and *Moringa stenopetala* in the tropical regions. All maps were generated by authors of this work using ArcGIS 10.8.2 (https://www.arcgis.com/index.html).

Currently *M. oleifera* is distributed over an area of 21.1 million km^2^ whereas *M. stenopetala* is distributed over 0.91 million km^2^ area. The current distribution of *M. oleifera* covers countries in Southeast Asia, Central Africa, Central America, South America, and Oceania (Fig. 1). On the other hand, *M. stenopetala* was chiefly distributed in its native range in Ethiopia and Kenya, and very few were recorded from Central America (Fig. 1). However, a few individuals of *M. stenopetala* were also reported from India. The ensemble model projections indicated high habitat suitability for *M. oleifera* in India, Ghana, Burkina Faso, Mexico, Parts of Venezuela, Colombia, and Australia. On the other hand, the model projected that only Ethiopia and parts of Kenya will be highly suitable for the future distribution of *M. stenopetala*. Under the different climate change scenarios, parts of Central America may also provide suitable areas for *M. stenopetala* but not *M. oleifera*.

**Figure 2:**
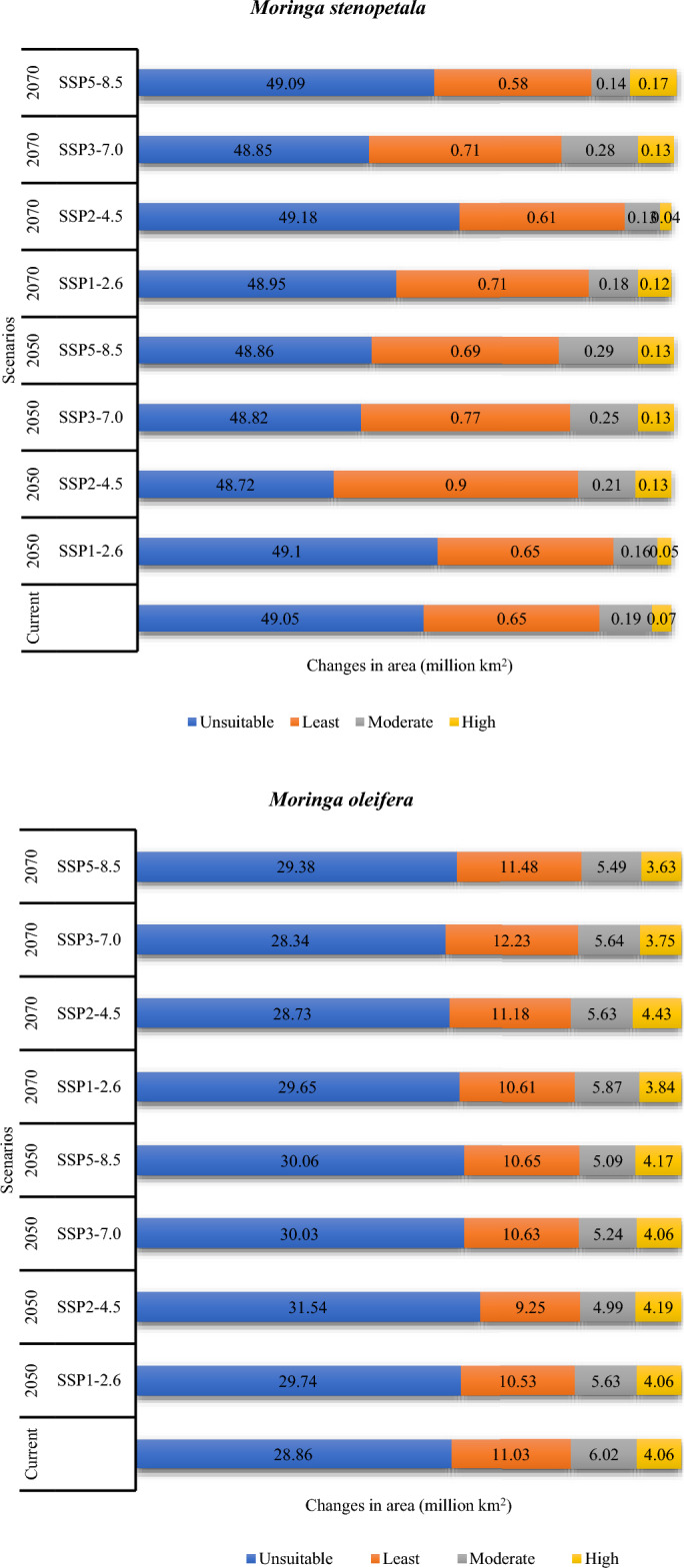
Changes in habitat suitability area in current and future (2050 and 2070) climate scenarios for *M. oleifera* and *M. stenopetala.*

**Figure 3:**
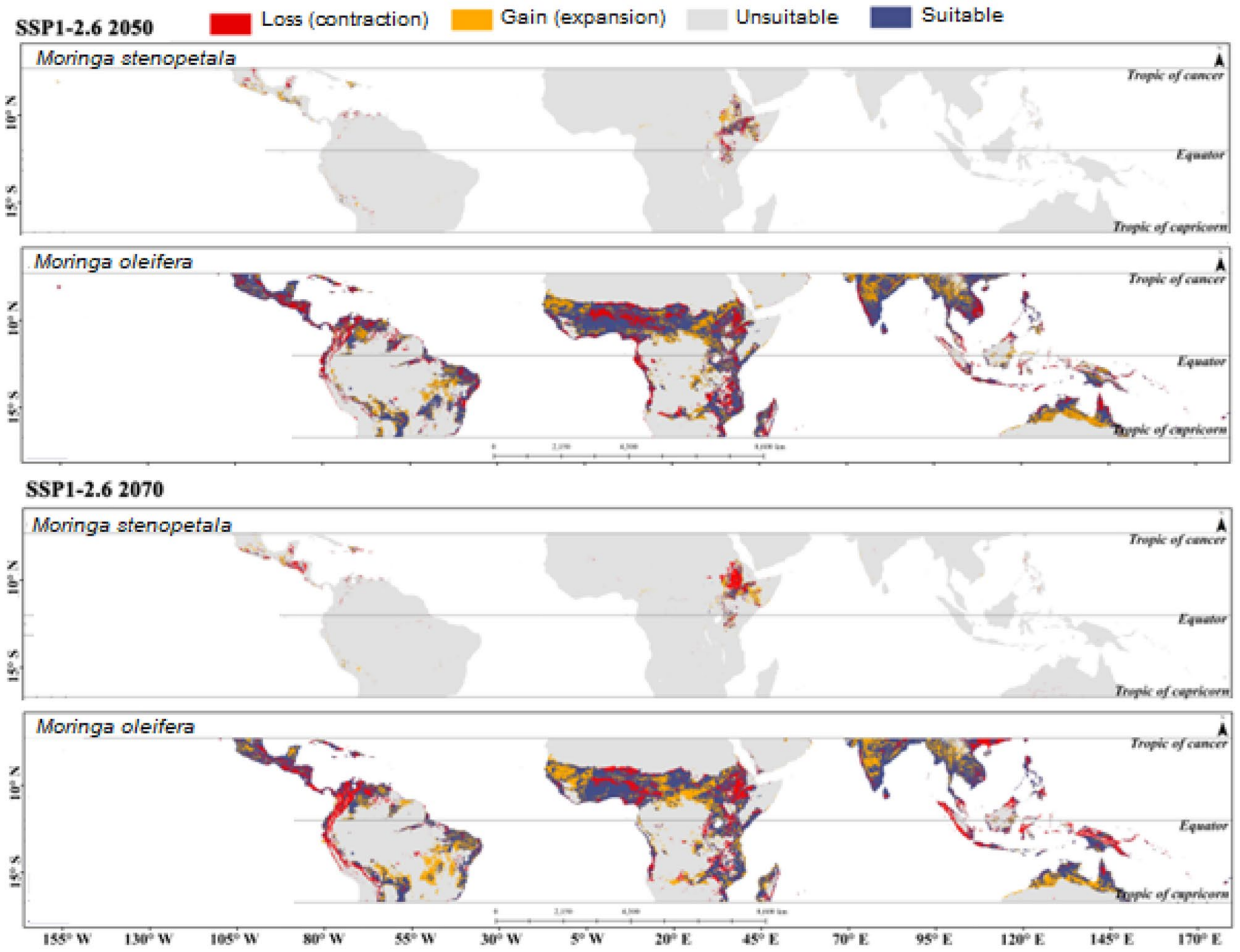
Changes in habitat suitability area of *Moringa oleifera* and *Moringa stenopetala* under SSP1-2.6 by 2050 (top) 2070 (bottom). All maps were generated by authors of this work using ArcGIS 10.8.2 (https://www.arcgis.com/index.html).

**Figure 4:**
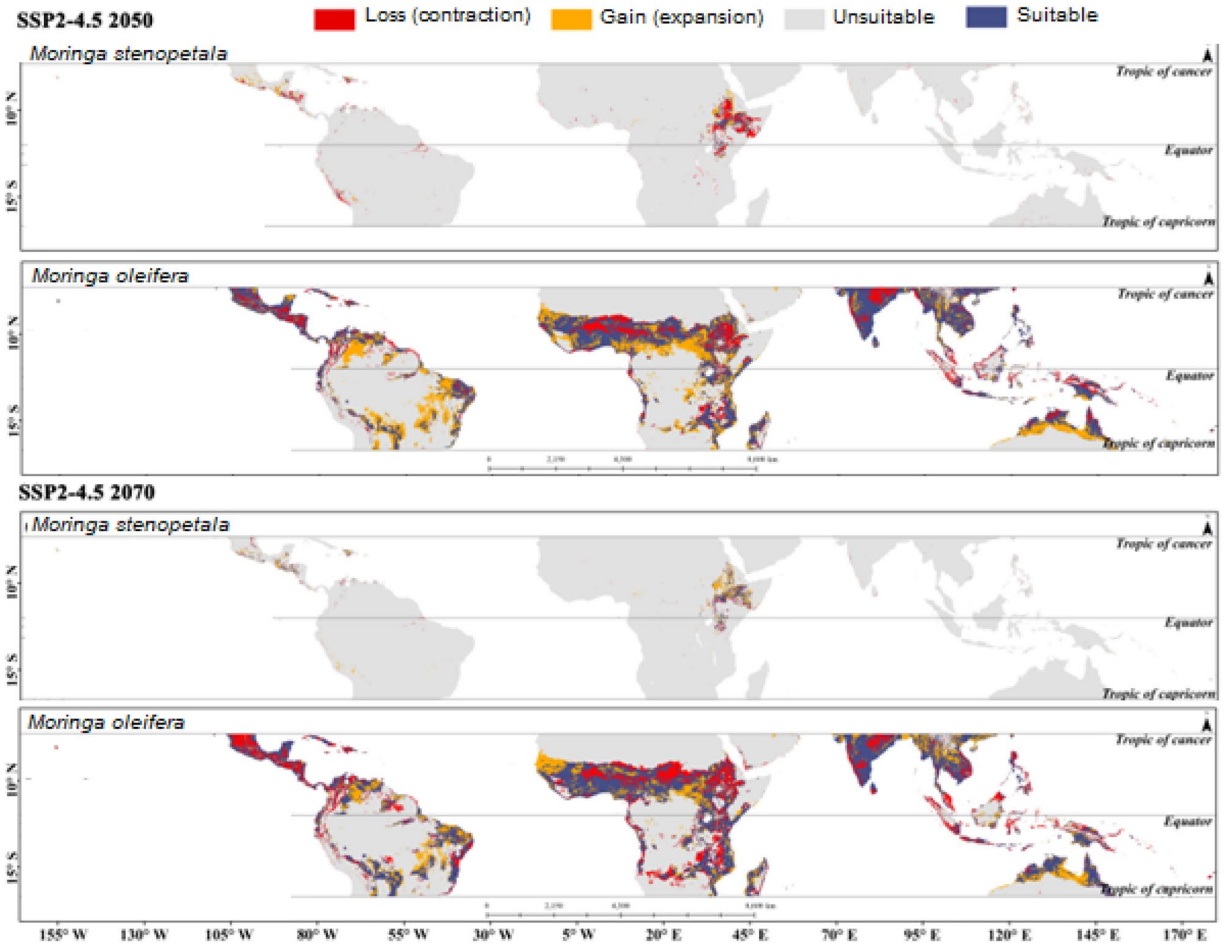
Changes in habitat suitability area of *Moringa oleifera* and *Moringa stenopetala* under SSP2-4.5 by 2050 (top) 2070 (bottom). All maps were generated by authors of this work using ArcGIS 10.8.2 (https://www.arcgis.com/index.html).

**Figure 5:**
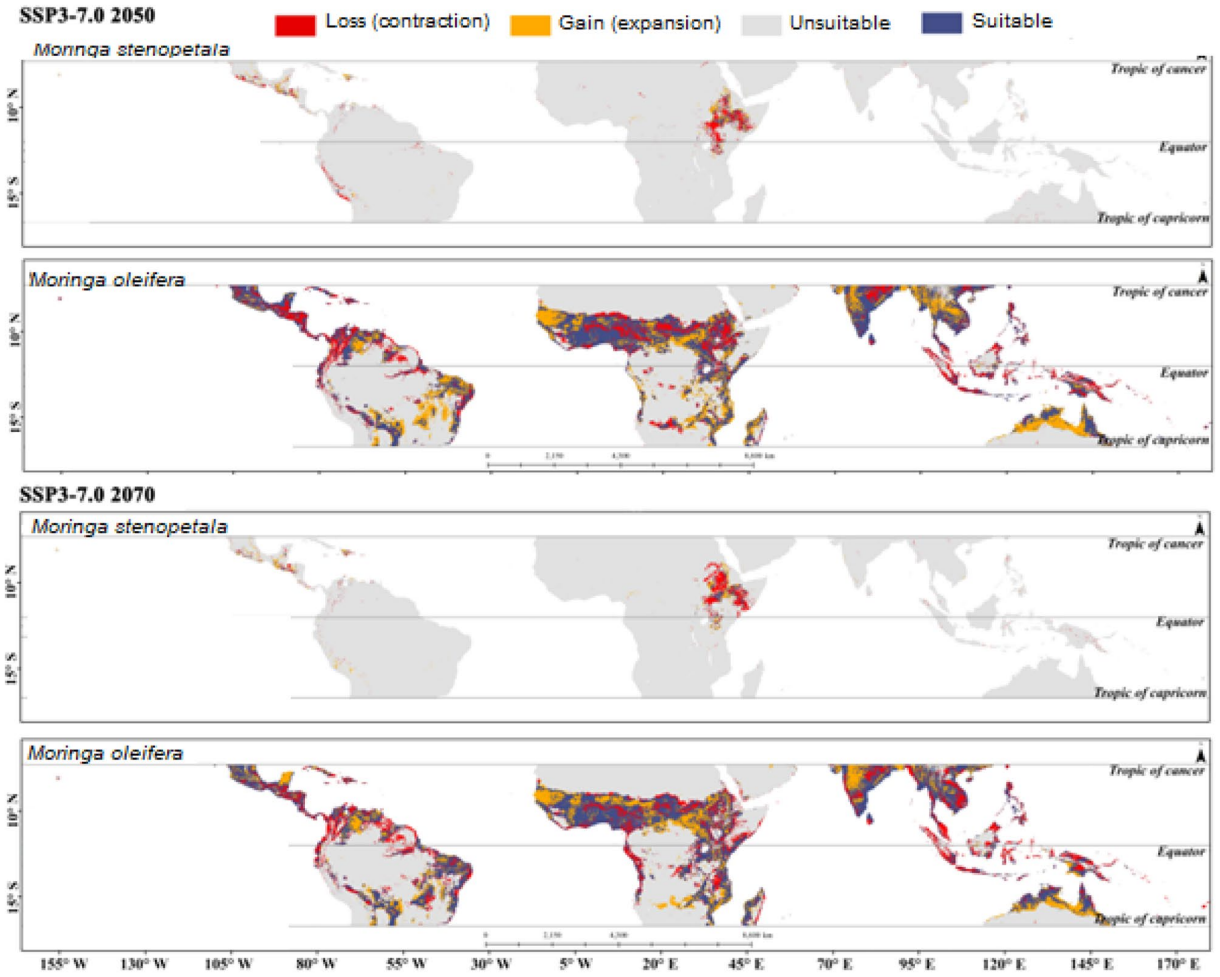
Changes in habitat suitability area of *Moringa oleifera* and *Moringa stenopetala* under SSP3-7.0 by 2050 (top) 2070 (bottom). All maps were generated by authors of this work using ArcGIS 10.8.2 (https://www.arcgis.com/index.html).

**Figure 6:**
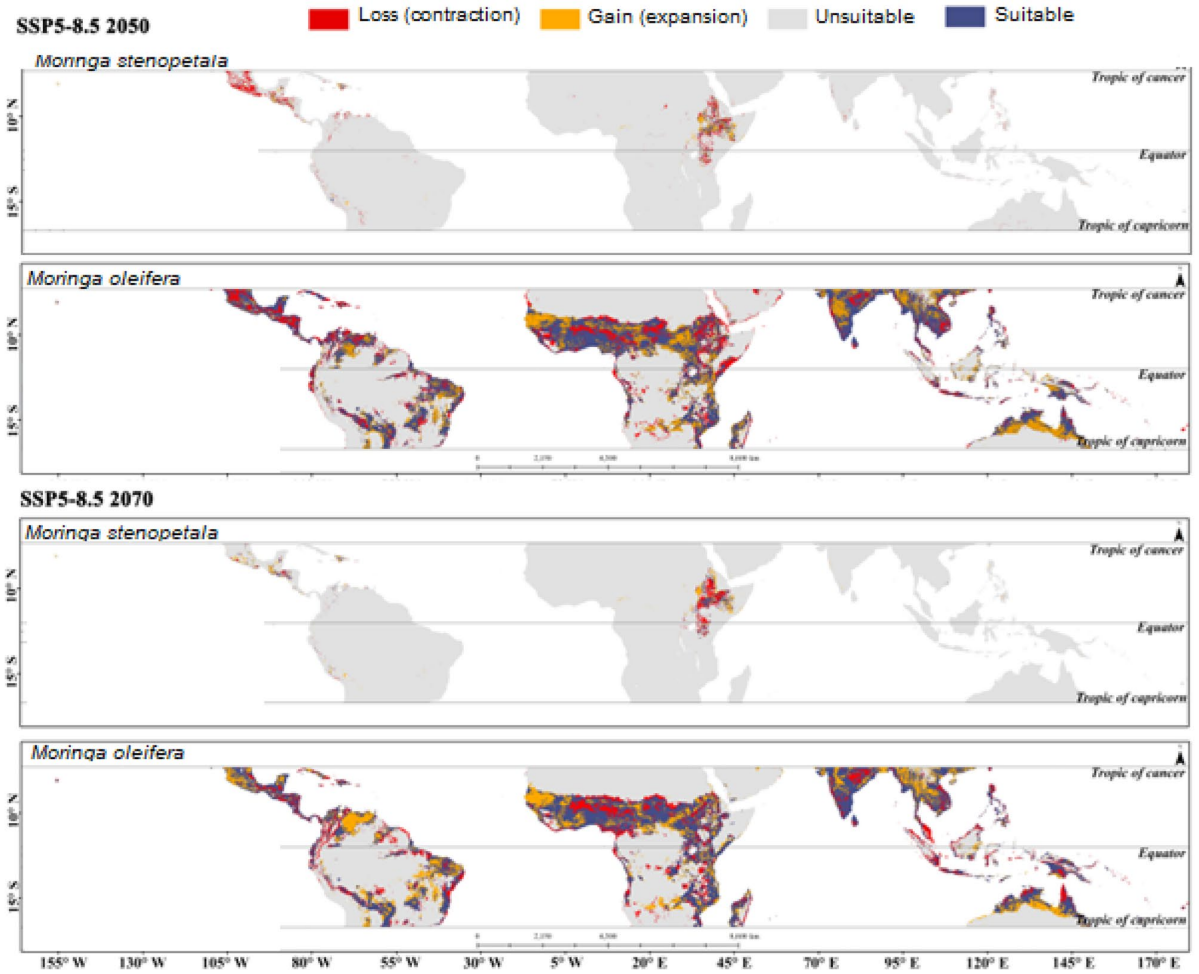
Changes in habitat suitability area of *Moringa oleifera* and *Moringa stenopetala* under SSP5-8.5 by 2050 (top) 2070 (bottom). All maps were generated by authors of this work using ArcGIS 10.8.2 (https://www.arcgis.com/index.html).

The estimated potentially suitable habitat assuming the different climate scenarios for *M. oleifera* and *M. stenopetala* in tropical countries is presented in Supplementary Figs. S1 to S4. Tropical countries cover a total area of about 49.97 million km^2^, of which 4.06 million km^2^ (8.12%) and 0.07 million km^2^ (0.13%) was projected to be highly suitable for *M. oleifera* and *M. stenopetala*, respectively, in the current scenario. In addition, 6.02 million km^2^ was predicted to be moderately suitable, 11.03 million km^2^ was least suitable, and 28.86 million km^2^ was unsuitable for *M. oleifera*. On the other hand, 0.02 km^2^, 0.65 million km^2^, and 49.05 million km^2^ were deemed to be moderately suitable, least suitable and unsuitable for *M. stenopetala* under the current climate scenario. The model predicted that about 4.06–4.19 million km^2^ by 2050 and 3.63‒4.43 million km^2^ area will be highly suitable for *M. oleifera* by 2070 under different climate scenarios. On the other hand, the highly suitable area for *M. stenopetala* was predicted to be about 0.05‒0.13 million km^2^ by 2050 and 0.04‒0.17 million km^2^ by 2070 (Fig. 2). Habitat suitability for both *M. oleifera* and *M. stenopetala* was projected to change under the different climate change scenarios (Figs. 3, 4, 5, 6). By 2050, the highly suitable area will expand by up to 3.2% for *M. oleifera* under SSP2-4.5. Under SSP2-4.5, the least suitable area would decrease by up to 16.2% by 2050. These areas will decrease by 3.9% under SSP1-2.6, but under SSP2-4.5, SSP3-7.0 and SSP5-8.5 this area will increase from 1.3 to 10.9% by 2070. Under SSP2-4.5, SSP3-7.0 and SSP5-8.5, the overall habitat suitability area for *M. stenopetala* is projected to increase from 5.6 to 97.9%.

### Niche overlap and distribution area for both species

**Figure 7:**
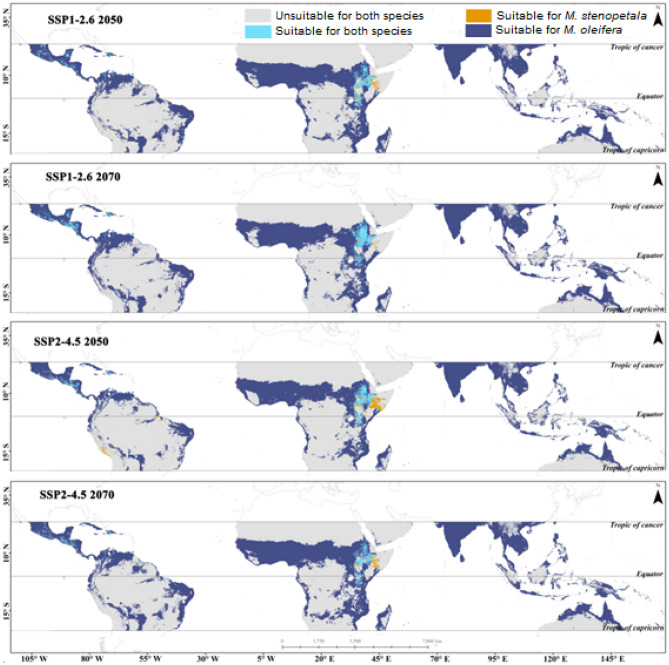
Changes in the overlapping area of *Moringa oleifera* and *Moringa stenopetala* under SSP1-2.6 and SSP2-4.5 by 2050 and 2070. All maps were generated by authors of this work using ArcGIS 10.8.2 (https://www.arcgis.com/index.html).

**Figure 8:**
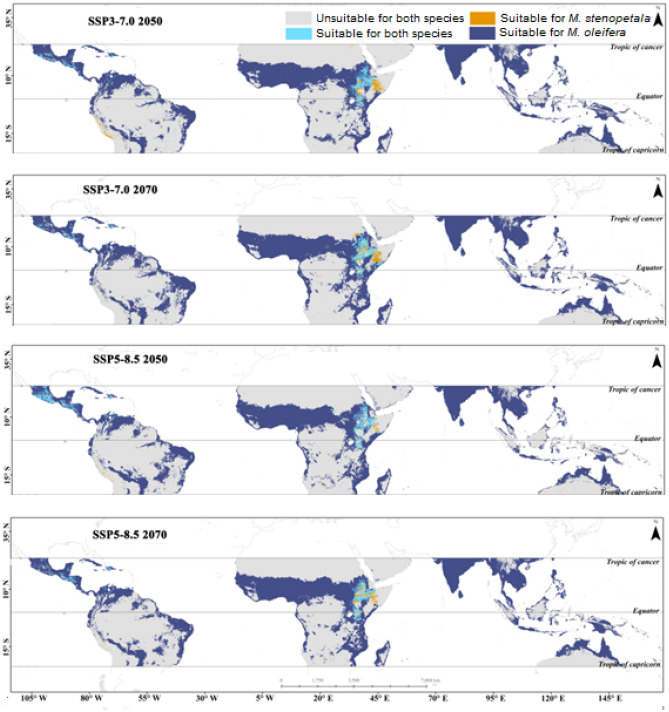
Changes in the overlapping area of *Moringa oleifera* and *Moringa stenopetala* under SSP3-7.0 and SSP5-8.5 by 2050 and 2070. All maps were generated by authors of this work using ArcGIS 10.8.2 (https://www.arcgis.com/index.html).

According to the ensemble model projection, the degree of niche overlap of *M. oleifera* and *M. stenopetala* was low in the suitable areas under the current climatic scenario. However, it is projected to increase in future epochs under SSP1-2.6. Under SSP2-4.5 and SSP3-7.0, the niche overlap is projected to decrease over time. The niche overlap is expected to increase under SSP2-4.5 by 2070. In the SSP5-8.5 scenario, the niche overlap of the suitable area is projected to increase significantly (Table 2). The suitable area for both species was found in parts of Africa, specifically in Ethiopia, Kenya, and some parts of Sudan, with overlapping areas increasing in the future under SSP1-2.6 2050, SSP2-4.5 2050, and SSP5-8.5 2050. However, the overlapping area is projected to decrease under SSP3-7.0 2050. Furthermore, the overlapping area is expected to increase under the SSP1-2.6 scenario for 2070, but decrease under SSP2-4.5, SSP3-7.0, and SSP5-8.5 (Figs. 7, 8). Areas where significant overlap is projected to occur by 2070 are mainly driven by soil salinity, water holding capacity, slope, isothermality, and mean temperature of the driest quarter.

**Table 2:** Ecological niche overlap of suitable habitat between *M. oleifera* and *M. stenopetala.*

Niche overlap	Schoener’s parameter (D)	Hellinger’s-based parameter (I)
Current	0.48	0.79
SSP1-2.6 2050	0.55	0.83
SSP2-4.5 2050	0.44	0.75
SSP3-7.0 2050	0.42	0.74
SSP5-8.5 2050	0.43	0.75
SSP1-2.6 2070	0.54	0.83
SSP2-4.5 2070	0.56	0.84
SSP3-7.0 2070	0.40	0.72
SSP5-8.5 2070	0.45	0.76

### Relative variable importance

**Figure 9:**
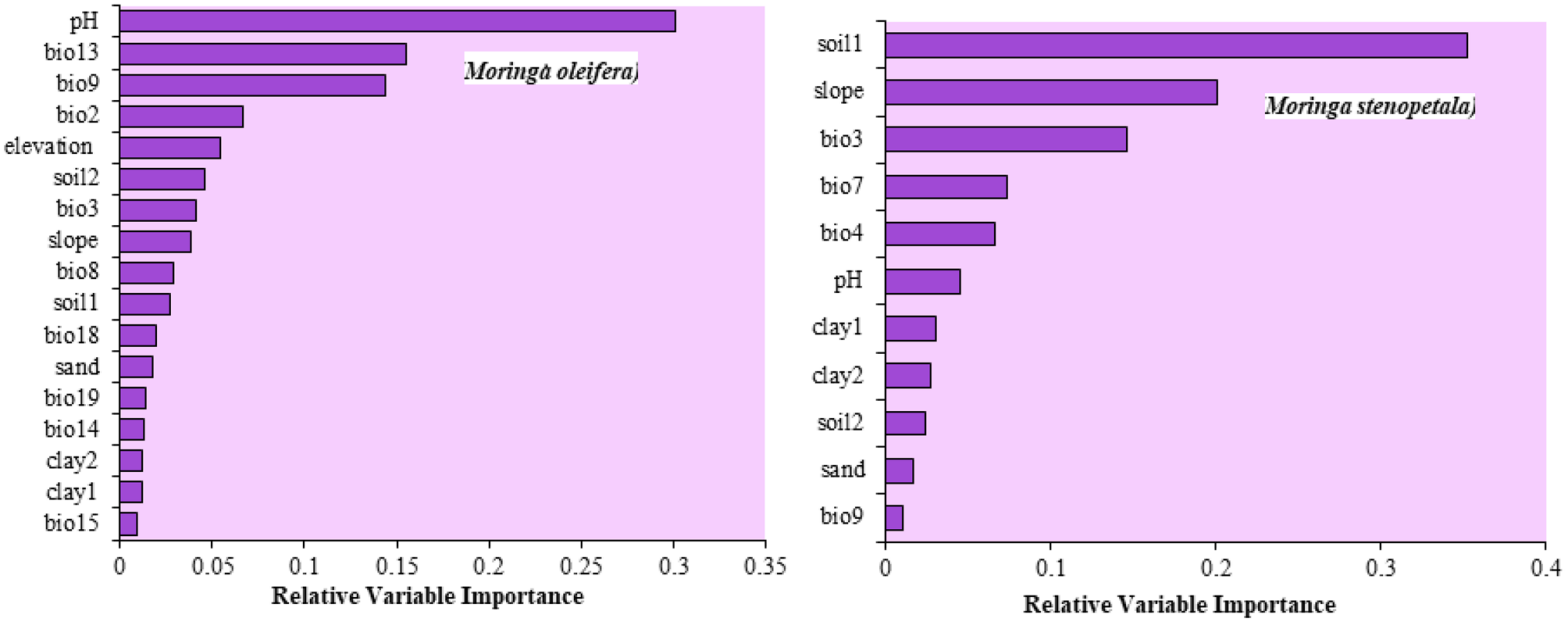
Relative variable importance of *Moringa oleifera* and *Moringa stenopetala*. Soil1 = soil salinity, soil2 = water holding capacity, pH = soil pH, clay1 = , clay2 = , bio2 = Mean Diurnal Range, bio3 = Isothermality, bio4 = Temperature Seasonality, bio7 = Temperature Annual Range, bio8 = Mean Temperature of Wettest Quarter, bio9 = Mean Temperature of Driest Quarter, bio13 = Precipitation of Wettest Month, bio14 = Precipitation of Driest Month, bio15 = Precipitation Seasonality, bio18 = Precipitation of Warmest Quarter, bio19 = Precipitation of Coldest Quarter.

In the ensemble model, the mean temperature of the driest quarter (Bio 9), precipitation of the wettest month (Bio 13), and mean diurnal temperature range (Bio 2) were the most influential bioclimatic variables in the potential distribution of *M. oleifera*. Isothermality (Bio 3), annual temperature range (Bio 7), and temperature seasonality (Bio 4) were highly influential in the case of *M. stenopetala* (Fig. 9). Among soil variables, soil pH, elevation and soil water holding capacity had higher influence on the distribution of *M. oleifera*, whereas soil salinity, slope, and soil pH were more influential for the current distribution of *M. stenopetala*. The clay and sand contents had the least contribution to the distribution of both *Moringa* species (Fig. 9).

Areas that are highly suitable for *M. stenopetala* were characterized by slightly saline soil of 1–2 dS m^−1^, a slope of 10–25°, isothermality of 60–90%, annual temperature range of 15–22 °C, and temperature seasonality of 10–20%. The highly suitable areas for *M. oleifera* were characterized by soil pH of 6–8, precipitation of wettest month of 150–300 mm, mean temperature of the driest quarter of 15–25 °C, mean diurnal temperature range of 8–15 °C, elevation of 100–3000 masl and water holding capacity of 20–50% (Table 3).

**Table 3:** Characterization of highly suitable areas for *M. oleifera* and *M. stenopetala*.

Species	Variables	Description	Range
*Moringa oleifera*	pH	Soil pH	6–8
	Bio 13	Precipitation of wettest month	150–300 mm
	Bio 9	Mean temperature of driest quarter	15–25 °C
	Bio 2	Mean diurnal range (mean of monthly (max temp − min temp))	8–15 °C
	Elv	Elevation	100–3200 masl
	WHC	Water holding capacity	20%-50%
*Moringa stenopetala*	soil salinity	Soil salinity	1–2 dS m^−1^
	slope	Slope	10°–25°
	Bio 3	Isothermality (BIO2/BIO7) (× 100)	60–90%
	Bio 7	Temperature annual range (BIO5–BIO6)	15–22 °C
	Bio 4	Temperature seasonality (standard deviation × 100)	10–20%

## Discussion

This study has established the current and future distribution of *M. stenopetala* and *M. oleifera*. The strength of this study lies in the use of an ensemble modelling approach to increase the accuracy of the predictions and the use of different performance metrics to assess the models’ performance^35^. The results have also pointed out areas which are likely to be affected in the event that they become invasive. In such cases, practitioners need to be prepared to manage invasions in areas identified as highly suitable.

The distribution of *M. oleifera* and *M. stenopetala* is mainly concentrated in the West and East African countries like Kenya, Uganda, Ethiopia, parts of Sudan, and in parts of North American countries like south of Mexico, Guatemala. In contrast, the distribution of the *Moringa* tree is limited in countries like Botswana, Namibia, South Africa, Swaziland, and Zambia^36^, although the extent of suitable areas identified in these countries by our modelling was large.

The larger areas covered by *M. oleifera* may be explained by its fast growth and adaptation to a wide range of climatic and soil conditions. *M. oleifera* takes short time to first flowering, which is about 11 months from planting^37^ compared to the 2–2.5 years taken by *M. stenopetala* from planting to first flowering^37^. In addition, *M. oleifera* has been shown to withstand a wide range of precipitation conditions (annual rainfall of 250‒3000 mm)^17^. On the other hand, the ideal range of rainfall required for the growth of *M. stenopetala* is between 500 and 1400 mm^38^. *M. oleifera* is also adapted to a wide range of elevations (0‒2000 masl)^6^ compared to *M. stenopetala* (400‒1200 m)^39^. Our models have predicted that *M. oleifera* can occur at the elevation of 100‒3000 masl. Furthermore, *M. oleifera* can tolerate and grow well under temperatures ranging between 12.6 and 40 °C, while *M. stenopetala* grows better where temperatures are 24‒30 °C. *M. oleifera* grows better in well-drained clay or clay loam soils without prolonged waterlogging^38^. While *M. oleifera* can grow in soil with a wide pH range (5‒9), *M. stenopetala* mostly grows in soils with neutral reaction^38,40^.

Our model predicted that the overall suitable area of *M. oleifera* will be reduced, although some expansion may occur by 2070. The habitat suitability of *M. stenopetala* will decrease under SSP1-2.6, and only Ethiopia and parts of Kenya will be highly suitable for its future distribution. This is because some regions are projected to experience a decrease in temperature and increases in precipitation. These changes may result in a reduction in the area suitable for some species, particularly those adapted to warmer and drier conditions. This is because the cooler and wetter conditions will become more favourable for other species to thrive, displacing species adapted to warmer and drier conditions. As a result, suitable areas for some species may shrink. *M. stenopetala* appears to be favoured by future climate change scenarios as the highly suitable area tended to increase substantially by 2050 and 2070 under SSP5-8.5. SSP5-8.5 is expected to lead to increased variability and extreme events. These changes may result in an expansion of suitable areas for some species, particularly those adapted to warmer conditions. Similarly, a study on the habitat suitability of *Ficus squamosa* and *Ficus heterostyla* revealed a decline in their overlapping areas. However, among the two species, *F. heterostyla* demonstrated a greater potential for climate change adaptability. This confirms that many species may detect climatic changes and respond in various ways^41^.

Our results showed relatively high current and future overlap in the distributions of *M. oleifera* and *M. stenopetala*. Several factors favour the overlapping of both the *Moringa* species. Slightly saline soil of 1–2 dS m^−1^, water holding capacity of 20–40%, a slightly sloppy area between 10°–30°, isothermality between 65–90% and mean temperature of driest quarter of 20–35 °C are some of the favourable factors for the growth of both *M. oleifera* and *M. stenopetala*. As a consequence, the overlapping area between *M. oleifera* and *M. stenopetala* may tend to increase under current and future climate conditions.

Our model predicted that the distribution of both species is influenced by multiple factors like soil pH, soil salinity, elevation etc., which is in agreement with previous reports. A recent study has reported that elevation is one of the factors affecting the distribution of *Moringa* species, as it directly or indirectly influences the temperature and soil characteristics^42^. Additionally, the slope of a site was a very influential factor in the distribution of *Moringa* species. The majority of *Moringa* habitat was found on gravelly slopes and rocky mountainous areas with a slope of 40–60% indicating that they prefer well-drained soils^42^. Soil texture, bulk density, and soil porosity are the controlling factors affecting the WHC of the soil^43^, and the WHC of soil can affect the growth of plant species. Although *M. oleifera* is a drought tolerant tree, WHC was one of the significant factors affecting its growth. *M. oleifera* can grow well on soil having more than 70% WHC compared to soil having a lower WHC^44^. Our result revealed that WHC between 20–50% can favour the growth of *M. oleifera.* In changing climatic conditions, temperature and precipitation may severely alter the habitat suitability of these species.

The current distribution of *M. oleifera* and *M. stenopetala* was also highly influenced by soil pH and soil salinity. Soil pH in the natural environment has an enormous influence on the soil biological, chemical and physical properties that influences plant growth^45^. In our analysis, soil having a pH of 6.5–8 had the highest contribution in the current distribution of *M. oleifera*. However, previous studies have suggested that the *Moringa* tree can grow well in soil with pH between 4.5 to 8.5; and pH between 6.3 to 7 can improve the growth of the *Moringa* tree^33,46,47^. Soil salinity hinders plant growth by lowering leaf water potential, causing physiological and morphological alterations, producing reactive oxygen species, raising osmotic stress and ion toxicity, and changing biochemical processes^48^. However, it was reported that the *Moringa* trees can grow well and germinate at lower soil salinity^49^ of 5 dS m^−1^.

Our results indicate that currently suitable areas may likely become unsuitable for the growth of *M. oleifera* and *M. stenopetala* in future climate change scenarios. A similar finding was also reported for a economically important plant *Pinus gerardiana*. Where the models predicted a remarkable decline in the potential habitat suitability in the future climate change scenarios^50^. Climate change influences the physiochemical, and biological properties of soil which relates to the functional properties of soil. Drivers of climate change affects the organic matter status, carbon and nutrient cycling, plant available water, and hence productivity, which in turn affect soil pH^51^. Furthermore, due to climate change, there will be spatial and temporal changes in temperature and rainfall^52^. These temperature changes may affect evapotranspiration, including the evaporation of water from soils. As a result, the salinity of the soil will increase that will hinder the growth of plants^53^. Additionally, erratic rainfall can greatly affect the groundwater table and soil salinity. Lower level of groundwater table can instigate capillary rise which causes the upward movement of salts from the water table to the soil surface, resulted in the accumulation of salinity at or near the soil surface, and may cause salinity stress in the plants^42^. Moreover, extreme events of anthropogenic climate change can also alter the WHC of soil; the WHC may decrease in warmer climate in the future^54^. This may possess a severe impact on the distribution of the species because WHC is an important factor for plant growth and can compensate for a lack of precipitation in dry years^43^.

The results of this study have significant implications for research and development. The baseline information on the current distribution will be helpful to guide future research on the two species. In terms of development, the information provided on habitat suitability would be useful in guiding the targeting of the species to areas where they can be successfully introduce. Several international organizations, governments, and NGOs are making efforts to popularise these species in different agroforestry interventions to improve productivity and human nutrition^55^. Governments of several developing countries like Ghana, Cuba, and India have focused on *Moringa* for combating malnutrition and encouraging its cultivation^56^. However, identifying suitable areas for the plantation and growth is crucial. Output of the present study can be considered for a more effective implementation of these policies through promotion of these species in in areas suitable for their planting. The *Moringa* trees can act as a good carbon sink, as they produces heavy flushes even during the dry season, and can help in reducing the level of atmospheric carbon dioxide (CO_2_)^57^. Furthermore, the *Moringa* tree can absorb fifty times more CO_2_ compared to the Japanese cedar tree dominated vegetation and twenty times more CO_2_ than other vegetation^58^. In future climate change scenarios, if the highly suitable area for *Moringa* trees increases, it will also help in the adaptation of these trees for their various beneficial properties. However, to achieve the different goals, these *Moringa* species need to be planted in suitable niches with careful consideration of their potential for invasivenes. We recommend concerned bodies to promote the species in suitable areas to guarantee success.

## Conclusions

Using an ensemble model, we predict the current distribution and future habitat suitable for *M. oleifera* and *M. stenopetala*. The analysis has provided evidence that ensemble models can accurately predict the distributions of both *M. oleifera* and *M. stenopetala.* It is concluded that the habitat that is highly suitable for *M. stenopetala* will increase under future climate change scenarios. In contrast, the overall suitable area for *M. oleifera* will be reduced. Nevertheless, some expansion in the suitable area is likely to occur by 2070. It is also concluded that soil pH and water holding capacity were the major predictors of the current distribution of *M. oleifera*, whereas soil salinity, elevation and slope are key predictors of the current distribution of *M. stenopetala*. This study has provided baseline information on the current distribution and possible future habitat suitability. The results are hoped to help researchers, policymakers and practitioners to make informed choices when selecting areas for the promotion of these species under current and future climate change scenarios. Future research should aim to produce fine-scale climate projections that account for regional changes in temperature, precipitation, and extreme weather events. This would enable more precise estimation of viable habitats under various climate change scenarios. In addition, dynamic modelling and multi-species modelling of *Moringa* species with other species will also be beneficial in understanding the migration and adaptation patterns of *Moringa* species and how their habitat suitability interacts with other plant species. The maps we provided are also hoped to help in identifying appropriate niches for the cultivation of *Moringa* species for greater production.

## Materials and methods

### Study area

The present study covers tropical regions (from the Tropic of Cancer to the Tropic of Capricorn), comprising 122 countries. Of these, 51 are from Africa, 19 are Asian, 27 are North American, 13 are Oceanian, and 12 are from South America. This region accounts for about 36% of the earth’s landmass, and the mean annual temperature in the tropics ranges from 25 to 28 °C^59^.

### The target species

*Moringa oleifera* is commonly known as a *‘drumstick tree’* or ‘*horse-radish tree.*’ *Moringa oleifera* is a fast-growing, drought-resistant, deciduous, dicotyledonous tree with a height of 5–10 m^60^. *Moringa oleifera* can grow well in the humid tropics and hot, dry lands^11^ and endure a range of rainfall from 250–3000 mm and a pH of 5–9^61^. This species can be found at 0‒1000 masl elevation and can be adapted to various soil types^38^. The tree has a soft trunk, gummy bark, and a tripinnately compound leaf^62^.

*Moringa stenopetala* is popularly known as the African cabbage tree, a strongly branched tree with a thick base with white to pale grey or silvery bark. Its trunk can grow up to 60 cm in diameter at its breast, and the tree has smooth wood and soft leaves^63^. In Africa, *Moringa stenopetala* naturally grows with the *Acacia tortilis*–*Delonix elata*–*Commiphora* spp. vegetation complex and can be found at an altitude of 400–2100 m. *M. stenopetala* has no specific soil requirement for its growth^38^.

### Geo-location data

The data on the geo-locations of *M. oleifera* and *M. stenopetala* was collected from Global Biodiversity Information Facility (GBIF)^64^ and published in peer-reviewed literature (Fig. 1a; Supplementary Tables S1 and S2). The GBIF is an international organisation that focuses on making scientific data on biodiversity available via the Internet using web services.

The literature survey was carried out using the search engine “Google Scholar”. Search strings were created comprising the keywords “*Moringa oleifera*,” “*Moringa stenopetala*,” “drumstick tree,” “horse-reddish tree,” individually or in various combinations such as “*Moringa oleifera* in Asia” or “Distribution of *Moringa stenopetala”.* Deka et al*.*^65^ used similar data curation methods to examine the possible effects of climate change on the distribution of the endangered white-winged wood duck (*Asarcornis scutulata*, 1882) in the Indian Eastern Himalayan region. A total of 1739 (n = 133 literature, n = 1606 GBIF) locations were collected, out of which 1692 were for *M. oleifera,* and 47 were for *M. stenopetala* which were considered for niche modelling. However, to prevent the model from over-fitting, the numerous present points were removed from one grid cell^66^. The spThin^67^ package was used in the R software version 4.1.3 (R Core Team, 2022). Eventually, 652 occurrence records of *M. oleifera* (n = 100 literature, n = 552 GBIF) and 43 occurrence records of *M. stenopetala* (n = 33 literature, n = 10 GBIF) were considered to build the models. These are listed in Supplementary Table S1.

### Environmental data

The standard 19 bioclimatic variables and elevations were downloaded from WorldClim version 2.1^68^ with 30 Arc seconds, which are the average for the years 1970–2000^69^. These bioclimatic factors have often been utilized in SDMs for climate prediction based on tree species^70^. Moreover, 14 soil data layers were accessed from the International Soil Reference and Information Centre (https://www.isric.org)^71^. Slope data was created from the DEM in the ArcGIS spatial extension. The spatial resolution of all the environmental variables was 30 Arc seconds (~ 1 km). The complete list of variables are presented in Supplementary Table S2.

Among 35 variables (Table S2), a few (18 in *M. oleifera* and 24 in *M. stenopetala*) were filtered using variance inflation factor (VIF) to avoid the effect of multicollinearity (Supplementary Table S3). We used R package called “usdm” to calculate VIF. Values greater than VIF 10 signify that an unacceptably large quantity of collinearity has been eliminated^70^. Therefore, the modelling was performed using 17 variables for *M. oleifera* and 11 for *M. stenopetala*. Some variables were calculated from others. For example, mean diurnal temperature range was calculated as the difference between mean of monthly maximum temperature and minimum temperature.

### Future climate scenario data

To predict habitat suitability under future climate scenarios, we used the Coupled Model Intercomparison Project Phase 6 (CMIP6) downloaded from the WorldClim dataset. The CMIP6 is a recent climate projections data with the same spatial resolution of current period data from WorldClim for 2050 (average for 2041–2060), and 2070 (average for 2061–2080), under four shared socio‐economic pathway (SSP) scenarios (i.e., SSP1-2.6, SSP2-4.5, SSP3-7.0, and SSP5-8.5). Among these scenarios, SSP3-7.0 was the new scenario combinations, and the SSP1-2.6, SSP2-4.5 and SSP5-8.5 were the updated version of the RCP scenarios^71^. In this study, we exclude SSP4-6.0 as it has the highest inequality, followed by SSP3-7.0. Both SSP1-2.6 and SSP5-8.5 feature relatively equitable development and a rapid catch-up of the world’s poorest countries over the coming century^73^. SSP1-2.6 is the lower end of radiative forcing expected to produce < 2 °C warming, and for brevity here we will refer to it as “low” emission scenario. SSP2-4.5 represents the medium range of future pathways, and for brevity here, we will refer to it as “medium”. SSP5-8.5 represents the high end of the range of future pathways^72,73^, and here we refer to it as “high” climate change/emission scenario. SSP2-4.5 and SSP5-8.5 project global temperature anomalies of 2.4 °C and 4.9 °C above pre-industrial levels by 2100 with atmospheric CO_2_ equivalents of 650 and 1370 ppm, respectively^74,75^. Finally, the SSP dataset were used for model the *Moringa* sp. for future climate scenarios.

### Species distribution modelling

We applied an ensemble of six models implemented in the ‘sdm’ package^76^ in R (4.1.3 version). The models included one regression and five machine learning methods. We selected the multivariate adaptive regression splines (MARS) regression model and the support vector machine (SVM), boosted regression trees (BRT), random forest (RF), classification and regression tree (CART) and maximum entropy (MaxEnt) machine learning methods for their high predictive accuracy^77^.

The model was trained with 10 replicates and evaluated per algorithm through five cross-validations. The occurrence data were split into training (70%) and test (30%) data to explore the implications of different environmental variables. Presence–absence models used equal numbers of presences and pseudo-absences, within the grid cell around presence locations^78^. Model accuracy and validation were judged by the True Skill Statistic (TSS) and the area under the curve (AUC). AUC values typically range from 0 to 1, with values closer to 1 indicating a more potent model^79,80^; AUC values < 0.7 are considered poor, 0.7–0.9 moderate, and > 0.9 good^81,82^. In contrast, TSS is a threshold-dependent measure of model accuracy, with values of + 1 indicating complete agreement between predictions and observations and 0 or below indicating agreement no better than random classification^83,84^. TSS value was classified as poor (< 0.40), fair (0.40–0.55), good (0.55–0.70), very good (0.70–0.85), excellent (0.85–0.99) and perfect (0.99–1.0)^85,86^. The Pearson correlation coefficient (COR) and deviance statistic were also considered to evaluate the model performance. COR estimates the correlation between continuous prediction with observation^87^, and the Deviance statistic calculates the deviation between observed and fitted values^88^.

Based on the potential species distribution habitat suitability index (0–1), the ensemble output maps were divided into four classes: unsuitable (0.00–0.2), least (> 0.2–0.40), moderate (> 0.4–0.6), and high potential (> 0.6–1.00)^89^. The reclassified tool in ArcGIS 10.8.2 was used to classify the ensemble maps. A field calculator was used to determine the raster area of each class of map (Count area × Area pixel/1,000,000). The relative variable relevance for each model’s present prospective *Moringa* distribution was established using the averaged variable response curve. The percentage contribution of each variable to the ensemble model was used to determine the variables' relative relevance. It draws attention to significant environmental factors that had a key role in shaping the geographic range of the species.

### Supplementary Information

Supplementary Information.

## Data Availability

Data are available upon reasonable request to the Corresponding Author. However, raw data are also attached as Supplementary Material.
