# Supplementary material for: Modelling habitat suitability for Moringa oleifera and Moringa stenopetala under current and future climate change scenarios
Source: Sci Rep. 2023 Nov 18;13:20221. doi: 10.1038/s41598-023-47535-5 (PMC10657390; doi:10.1038/s41598-023-47535-5)
Supplement: Supplementary file 1 — Supplementary Information. [file 41598_2023_47535_MOESM1_ESM.docx]

# **Supplementary Tables**

**Table S1:** Location of *M. oleifera* and *M. stenopetala* used in SDM

| **Sl. No.** | **Species** | **Location** | **Geo-Coordinates** | | **Country** | **Reference** |
| --- | --- | --- | --- | --- | --- | --- |
|  |  |  | **Latitude** | **Longitude** |  |  |
| 1 | *Moringa oleifera* | Yusufari | 13.057 | 11.001 | Nigeria | Popoola and Obembe (2013) |
| 2 | *Moringa oleifera* | Zhanjiang | 21.26021 | 110.50437 | China | Qi et al. (2016) |
| 3 | *Moringa oleifera* | Al-Qassim | 24.450111 | 46.142639 | Saudi Arabia | Chigurupati et al. (2022) |
| 4 | *Moringa oleifera* | Agaie | 9.050428 | 6.568197 | Nigeria | Njinga et al. (2015) |
| 5 | *Moringa oleifera* | Dormaa Ahenkro | 7.269583 | -2.849167 | Ghana | Tetteh et al. (2021) |
| 6 | *Moringa oleifera* | Berekum | 7.447767 | -2.578817 | Ghana | Tetteh et al. (2021) |
| 7 | *Moringa oleifera* | Sunyani | 7.315917 | -2.305933 | Ghana | Tetteh et al. (2021) |
| 8 | *Moringa oleifera* | Ejisu | 6.732717 | -1.473933 | Ghana | Tetteh et al. (2021) |
| 9 | *Moringa oleifera* | Kumasi | 6.690967 | -1.627317 | Ghana | Tetteh et al. (2021) |
| 10 | *Moringa oleifera* | Bekwai | 6.507667 | -1.539933 | Ghana | Tetteh et al. (2021) |
| 11 | *Moringa oleifera* | Tamale-Kumbungu Rd | 9.498367 | -0.899517 | Ghana | Tetteh et al. (2021) |
| 12 | *Moringa oleifera* | Tamale | 9.44935 | -0.86475 | Ghana | Tetteh et al. (2021) |
| 13 | *Moringa oleifera* | Tamale | 9.422217 | -0.852333 | Ghana | Tetteh et al. (2021) |
| 14 | *Moringa oleifera* | Dormaa East | 7.273683 | -2.80065 | Ghana | Tetteh et al. (2021) |
| 15 | *Moringa oleifera* | Kumasi | 6.6775 | -1.566333 | Ghana | Tetteh et al. (2021) |
| 16 | *Moringa oleifera* | Kumbungu | 9.590533 | -1.027783 | Ghana | Tetteh et al. (2021) |
| 17 | *Moringa oleifera* | Sirigu | 10.948364 | -0.941234 | Ghana | Ntibrey et al. (2020) |
| 18 | *Moringa oleifera* | Sirigu | 10.805783 | -0.873479 | Ghana | Ntibrey et al. (2020) |
| 19 | *Moringa oleifera* | Wakaso | 6.233333 | 14.483333 | Cameroon | Ngounouno et al. (2021) |
| 20 | *Moringa oleifera* | Wakaso | 6.233333 | 14.483333 | Cameroon | Ngounouno et al. (2021) |
| 21 | *Moringa oleifera* | Wakaso | 6.25 | 14.483333 | Cameroon | Ngounouno et al. (2021) |
| 22 | *Moringa oleifera* | Jahun, Jigawa | 12.1 | 9.416667 | Nigeria | Abubakar (2013) |
| 23 | *Moringa oleifera* | Gwandu, Kebbi | 12.266667 | 6.55 | Nigeria | Abubakar (2013) |
| 24 | *Moringa oleifera* | Kafanchan, Kaduna | 11.566667 | 8.3 | Nigeria | Abubakar (2013) |
| 25 | *Moringa oleifera* | Gezawa, Kano | 12.233333 | 8.066667 | Nigeria | Abubakar (2013) |
| 26 | *Moringa oleifera* | Tudunwada, Kano | 12.016667 | 8.566667 | Nigeria | Abubakar (2013) |
| 27 | *Moringa oleifera* | Dutsin-ma, Katsina | 12.45 | 7.483333 | Nigeria | Abubakar (2013) |
| 28 | *Moringa oleifera* | Kafur, Katsina | 11.65 | 7.7 | Nigeria | Abubakar (2013) |
| 29 | *Moringa oleifera* | Dogondaji, Sokoto | 12.1 | 6.316667 | Nigeria | Abubakar (2013) |
| 30 | *Moringa oleifera* | Goronyo, Sokoto | 13.45 | 5.666667 | Nigeria | Abubakar (2013) |
| 31 | *Moringa oleifera* | Numan, Adamawa | 9.433333 | 12.3 | Nigeria | Abubakar (2013) |
| 32 | *Moringa oleifera* | Jamaare, Bauchi | 11.633333 | 9.866667 | Nigeria | Abubakar (2013) |
| 33 | *Moringa oleifera* | Bauchi, Bauchi | 10.5 | 10 | Nigeria | Abubakar (2013) |
| 34 | *Moringa oleifera* | Gombe, Gombe | 10.283333 | 11.166667 | Nigeria | Abubakar (2013) |
| 35 | *Moringa oleifera* | Kaltingo, Gombe | 9.633333 | 11.083333 | Nigeria | Abubakar (2013) |
| 36 | *Moringa oleifera* | Malobe, Taraba | 7.783333 | 10.216667 | Nigeria | Abubakar (2013) |
| 37 | *Moringa oleifera* | Mamado, Yobe | 11.7 | 11.083333 | Nigeria | Abubakar (2013) |
| 38 | *Moringa oleifera* | Damaturu, Yobe | 12.883333 | 11.216667 | Nigeria | Abubakar (2013) |
| 39 | *Moringa oleifera* | Benin City | 6.393729 | 5.620347 | Nigeria | Obiazikwor and Ojeile (2021) |
| 40 | *Moringa oleifera* | Punjab | 30.75 | 75.666667 | India | Sing et al. (2021) |
| 41 | *Moringa oleifera* | Rio Grande do Norte | -5.900133 | -35.357028 | Brazil | Silva et al. (2022) |
| 42 | *Moringa oleifera* | Islamabad | 33.693056 | 73.063889 | Pakistan | Javed et al. (2022) |
| 43 | *Moringa oleifera* | Benin City | 6.399821 | 5.609864 | Nigeria | Isikhuemen et al. (2020) |
| 44 | *Moringa oleifera* | Koramangala, Bangalore | 12.935193 | 77.624481 | India | Ravi et al. (2021) |
| 45 | *Moringa oleifera* | Somanahalli, Bangalore | 12.771683 | 77.505327 | India | Ravi et al. (2021) |
| 46 | *Moringa oleifera* | Nidaghatta, Bangalore | 12.620094 | 77.088356 | India | Ravi et al. (2021) |
| 47 | *Moringa oleifera* | Thuppinakkara, Mysure | 12.38108 | 76.44428 | India | Ravi et al. (2021) |
| 48 | *Moringa oleifera* | Mandya, Mysure | 12.29581 | 76.63938 | India | Ravi et al. (2021) |
| 49 | *Moringa oleifera* | Sulthanbathery, Wayandu | 11.662914 | 76.257022 | India | Ravi et al. (2021) |
| 50 | *Moringa oleifera* | Adivaram, Calicut | 11.192152 | 75.854918 | India | Ravi et al. (2021) |
| 51 | *Moringa oleifera* | Moozhikkal, Calicut | 11.295254 | 75.834512 | India | Ravi et al. (2021) |
| 52 | *Moringa oleifera* | Randathanil, Calicut | 10.962452 | 76.00521 | India | Ravi et al. (2021) |
| 53 | *Moringa oleifera* | Puduppadi, Calicut | 11.481156 | 75.975513 | India | Ravi et al. (2021) |
| 54 | *Moringa oleifera* | Panambra, Malappuram | 11.119453 | 75.892858 | India | Ravi et al. (2021) |
| 55 | *Moringa oleifera* | Mundanam, Malappuram | 11.067024 | 76.089951 | India | Ravi et al. (2021) |
| 56 | *Moringa oleifera* | Irinjalakkuda, Trissur | 10.346892 | 76.207412 | India | Ravi et al. (2021) |
| 57 | *Moringa oleifera* | Alathur, Palakkadu | 10.645397 | 76.645397 | India | Ravi et al. (2021) |
| 58 | *Moringa oleifera* | Chittoor, Palakkad | 11.062395 | 76.654266 | India | Ravi et al. (2021) |
| 59 | *Moringa oleifera* | Ernakulam | 9.981636 | 76.299884 | India | Ravi et al. (2021) |
| 60 | *Moringa oleifera* | Cherthala, Alleppy | 9.674136 | 76.340096 | India | Ravi et al. (2021) |
| 61 | *Moringa oleifera* | Konni, Pathanamthitta | 9.226706 | 76.849678 | India | Ravi et al. (2021) |
| 62 | *Moringa oleifera* | Mundakkal, Kollam | 8.865983 | 76.6095 | India | Ravi et al. (2021) |
| 63 | *Moringa oleifera* | Attingal, Thiruvananthapuram | 8.698223 | 76.813657 | India | Ravi et al. (2021) |
| 64 | *Moringa oleifera* | Nagercoil, Kanyakumari | 8.183286 | 77.4119 | India | Ravi et al. (2021) |
| 65 | *Moringa oleifera* | Ukkadam, Coimbatore | 10.990213 | 76.962866 | India | Ravi et al. (2021) |
| 66 | *Moringa oleifera* | Hosur, Krishnagiri | 12.740913 | 77.825292 | India | Ravi et al. (2021) |
| 67 | *Moringa oleifera* | Tura, Meghalaya | 25.41445 | 90.21975 | India | Shankar et al. (2015) |
| 68 | *Moringa oleifera* | Ga-Masemola | -24.559583 | 29.630028 | South Africa | Fosso-Kankeu et al. (2019) |
| 69 | *Moringa oleifera* | Maiduguri metropolis | 11.829286 | 13.156427 | Nigeria | Arku and Musa (2021) |
| 70 | *Moringa oleifera* | Cheranchi | 12.669444 | 7.734167 | Nigeria | Yaradua and Amenu (2022) |
| 71 | *Moringa oleifera* | Hawassa | 7.052403 | 39.485591 | Ethiopia | Melesse et al. (2012) |
| 72 | *Moringa oleifera* | Arbaminch | 6.020605 | 37.564109 | Ethiopia | Melesse et al. (2012) |
| 73 | *Moringa oleifera* | Arbaminch | 6.033056 | 37.549722 | Ethiopia | Mikore and Mulugeta (2017) |
| 74 | *Moringa oleifera* | Konso | 5.25 | 37.483056 | Ethiopia | Mikore and Mulugeta (2017) |
| 75 | *Moringa oleifera* | Sonitpur, Assam | 26.676987 | 92.698379 | India | Saha et al. (2015) |
| 76 | *Moringa oleifera* | Karbi Anglong, Assam | 26.186085 | 93.581269 | India | Kumar et al. (2020) |
| 77 | *Moringa oleifera* | Lakhimpur, Assam | 27.206363 | 94.151327 | India | Kalita and Borah (2007) |
| 78 | *Moringa oleifera* | Cachar, Assam | 24.683334 | 92.683334 | India | Kalita and Borah (2007) |
| 79 | *Moringa oleifera* | Khulhat Reserve Forest, Assam | 26.0994 | 92.320226 | India | Hazarika and Khanikor (2022) |
| 80 | *Moringa oleifera* | Golaghat, Assam | 26.362391 | 93.862221 | India | Soren et al. (2021) |
| 81 | *Moringa oleifera* | Balakot | 34.548152 | 73.353185 | Pakistan | Iqbal and Bhanger (2006) |
| 82 | *Moringa oleifera* | Chakwal | 32.932788 | 72.862958 | Pakistan | Iqbal and Bhanger (2006) |
| 83 | *Moringa oleifera* | Mardan | 34.198639 | 72.040429 | Pakistan | Iqbal and Bhanger (2006) |
| 84 | *Moringa oleifera* | Nawabshah | 26.244697 | 68.393549 | Pakistan | Iqbal and Bhanger (2006) |
| 85 | *Moringa stenopetala* | Derashe | 5.590478 | 37.211583 | Ethiopia | Bedane et al. (2013) |
| 86 | *Moringa stenopetala* | Arbaminch Zuria | 6.0196 | 37.549929 | Ethiopia | Bedane et al. (2013) |
| 87 | *Moringa stenopetala* | Konso | 5.340499 | 37.438789 | Ethiopia | Bedane et al. (2013) |
| 88 | *Moringa stenopetala* | Derashe | 5.590478 | 37.211583 | Ethiopia | Meskel et al. (2020) |
| 89 | *Moringa stenopetala* | Konso | 5.340499 | 37.438789 | Ethiopia | Meskel et al. (2020) |
| 90 | *Moringa stenopetala* | Gamo Gofa | 6.360907 | 37.125859 | Ethiopia | Ayube et al. (2003) |
| 91 | *Moringa stenopetala* | Hawassa | 7.052403 | 39.485591 | Ethiopia | Melesse et al. (2012) |
| 92 | *Moringa stenopetala* | Arbaminch | 6.020605 | 37.564109 | Ethiopia | Melesse et al. (2012) |
| 93 | *Moringa stenopetala* | Hawassa | 7.066667 | 38.516667 | Ethiopia | Gebregiorgis et al. (2012) |
| 94 | *Moringa stenopetala* | Arbaminch | 6.014528 | 37.549824 | Ethiopia | Mekonnen et al. (1999) |
| 95 | *Moringa stenopetala* | Hawassa | 7.050374 | 38.495504 | Ethiopia | Melesse et al. (2008) |
| 96 | *Moringa stenopetala* | Gamo Gofa | 6.355353 | 37.125519 | Ethiopia | Toma et al. (2014) |
| 97 | *Moringa stenopetala* | Arbaminch | 6.033056 | 37.549722 | Ethiopia | Mikore and Mulugeta (2017) |
| 98 | *Moringa stenopetala* | Konso | 5.25 | 37.483056 | Ethiopia | Mikore and Mulugeta (2017) |
| 99 | *Moringa stenopetala* | Hawassa | 7.083333 | 38.483333 | Ethiopia | Melesse et al. (2013) |
| 100 | *Moringa stenopetala* | Hawassa | 7.066667 | 38.516667 | Ethiopia | Melesse et al. (2011) |
| 101 | *Moringa stenopetala* | Nairobi | -1.294652 | 36.843492 | Kenya | Lalas et al. (2003) |
| 102 | *Moringa stenopetala* | Kottayam, Kerala | 9.569397 | 76.521917 | India | Habtemariam and Varghese (2015) |
| 103 | *Moringa stenopetala* | Gamo Gofa | 6.515693 | 36.954109 | Ethiopia | Toma et al. (2015) |
| 104 | *Moringa stenopetala* | Arbaminch | 6.031706 | 37.55157 | Ethiopia | Nardos et al. (2011) |
| 105 | *Moringa stenopetala* | Arbaminch | 6.033056 | 37.549722 | Ethiopia | Dadi et al. (2019) |
| 106 | *Moringa stenopetala* | Arbaminch | 6.424291 | 38.279022 | Ethiopia | Fereja et al. (2020) |
| 107 | *Moringa stenopetala* | Wolaita sodo town | 6.852809 | 37.760969 | Ethiopia | Tamrat et al. (2017) |
| 108 | *Moringa stenopetala* | Konso | 5.340449 | 37.438789 | Ethiopia | Mitiku and Yilma (2017) |
| 109 | *Moringa stenopetala* | Arbaminch | 6.065255 | 37.560112 | Ethiopia | Badessa et al. (2020) |
| 110 | *Moringa stenopetala* | Arbaminch | 6.042417 | 37.558792 | Ethiopia | Sileshi et al. (2014) |
| 111 | *Moringa stenopetala* | Gofa | 6.612078 | 37.166086 | Ethiopia | Haile et al. (2019) |
| 112 | *Moringa stenopetala* | Shella | 6.237764 | 37.281664 | Ethiopia | Haile et al. (2019) |
| 113 | *Moringa stenopetala* | Konso | 5.340449 | 37.438789 | Ethiopia | Haile et al. (2019) |
| 114 | *Moringa stenopetala* | Arbaminch | 6.065267 | 37.560113 | Ethiopia | Manilal et al. (2020) |
| 115 | *Moringa stenopetala* | Wolaitta zone | 6.856391 | 37.757949 | Ethiopia | Toma et al. (2012) |
| 116 | *Moringa stenopetala* | Ambo | 8.958061 | 37.932101 | Ethiopia | Raghavendra et al. (2016) |
| 117 | *Moringa stenopetala* | Kafta-Sheraro National Park | 14.250241 | 37.262077 | Ethiopia | Temesgen et al. (2021) |

Table S2: Initially define environmental variables for predicting distribution of *Moringa oleifera* and *Moringa stenopetala* in tropical region

| Variables | Description | Used in SDM | Species |
| --- | --- | --- | --- |
| Bio_1 | Annual Mean Temperature | × | - |
| Bio_2 | Mean Diurnal Range (Mean of monthly (max temp - min temp)) | ✓ | *M. oleifera* |
| Bio_3 | Isothermality (BIO2/BIO7) (×100) | ✓ | *M. oleifera / M. stenopetala* |
| Bio_4 | Temperature Seasonality (standard deviation ×100) | ✓ | *M. stenopetala* |
| Bio_5 | Max Temperature of Warmest Month | × | - |
| Bio_6 | Min Temperature of Coldest Month | × | - |
| Bio_7 | Temperature Annual Range (BIO5-BIO6) | ✓ | *M. stenopetala* |
| Bio_8 | Mean Temperature of Wettest Quarter | ✓ | *M. oleifera* |
| Bio_9 | Mean Temperature of Driest Quarter | ✓ | *M. oleifera / M. stenopetala* |
| Bio_10 | Mean Temperature of Warmest Quarter | × | - |
| Bio_11 | Mean Temperature of Coldest Quarter | × | - |
| Bio_12 | Annual Precipitation | × | - |
| Bio_13 | Precipitation of Wettest Month | ✓ | *M. oleifera* |
| Bio_14 | Precipitation of Driest Month | ✓ | *M. oleifera* |
| Bio_15 | Precipitation Seasonality (Coefficient of Variation) | ✓ | *M. oleifera* |
| Bio_16 | Precipitation of Wettest Quarter | × | - |
| Bio_17 | Precipitation of Driest Quarter | × | - |
| Bio_18 | Precipitation of Warmest Quarter | ✓ | *M. oleifera* |
| Bio_19 | Precipitation of Coldest Quarter | ✓ | *M. oleifera* |
| sand_0_5cm_mean | Sand (0-5cm) | × | - |
| sand_5_15cm_mean | Sand (5-15cm) | × | - |
| sand_15_30cm_mean | Sand (15-30cm) | × | - |
| sand_100_200cm_mean | Sand (100-200cm) | ✓ | *M. oleifera* / *M. stenopetala* |
| clay_0_5cm_mean | Clay (0-5cm) | ✓ | *M. oleifera* / *M. stenopetala* |
| clay_5_15cm_mean | Clay (5-15cm) | ✓ | *M. stenopetala* |
| clay_15_30cm | Clay (15-30cm) | × | - |
| clay_100_200cm_mean | Clay (100-200cm) | ✓ | *M. oleifera* / *M. stenopetala* |
| phh2o_0_5cm_mean | pH (0-5cm) | ✓ | *M. stenopetala* |
| phh2o_5_15cm_mean | pH (5-15cm) | × | - |
| phh2o_15_30cm_mean | pH (15-30cm) | × | - |
| phh2o_100_200cm_mean | pH (100-200cm) | ✓ | *M. oleifera* |
| Elv | Elevation | ✓ | *M. oleifera* |
| slope | Slope | ✓ | *M. oleifera* / *M. stenopetala* |
| whc_soil | Water holding capacity of soil | ✓ | *M. oleifera* / *M. stenopetala* |
| soil salinity | Soil salinity | ✓ | *M. oleifera* / *M. stenopetala* |

**Table S3:** Bioclimatic and biophysical variables for *M. oleifera* and *M. stenopetala* used in this study

| **Variables** | **Description** | **VIF** |
| --- | --- | --- |
| *M. oleifera* |  |  |
| Bio_13 | Precipitation of Wettest Month | 3.24 |
| Bio_14 | Precipitation of Driest Month | 2.67 |
| Bio_15 | Precipitation Seasonality (Coefficient of Variation) | 5.71 |
| Bio_18 | Precipitation of Warmest Quarter | 2.93 |
| Bio_19 | Precipitation of Coldest Quarter | 2.39 |
| Bio_2 | Mean Diurnal Range (Mean of monthly (max temp - min temp)) | 3.58 |
| Bio_3 | Isothermality (BIO2/BIO7) (×100) | 3.26 |
| Bio_8 | Mean Temperature of Wettest Quarter | 6.27 |
| Bio_9 | Mean Temperature of Driest Quarter | 4.57 |
| clay_0_5cm_mean | Clay (0-5cm) | 4.91 |
| clay_100_200cm_mean | Clay (100-200cm) | 5.12 |
| Elv | Elevation | 8.31 |
| phh2o_100_200cm_mean | pH (100-200cm) | 2.89 |
| sand_100_200cm_mean | Sand (100-200cm) | 5.82 |
| slope | Slope | 1.66 |
| whc_soil | Water holding capacity of soil | 1.08 |
| soil salinity | Soil salinity | 1.44 |
| *M. stenopetala* |  |  |
| Bio_3 | Isothermality (BIO2/BIO7) (×100) | 3.63 |
| Bio_4 | Temperature Seasonality (standard deviation ×100) | 3.33 |
| Bio_7 | Temperature Annual Range (BIO5-BIO6) | 4.01 |
| Bio_9 | Mean Temperature of Driest Quarter | 3.09 |
| clay_0_5cm_mean | Clay (0-5cm) | 3.38 |
| clay_100_200cm_mean | Clay (100-200cm) | 6.48 |
| phh2o_0_5cm_mean | pH (0-5cm) | 4.26 |
| sand_15_30cm_mean | Sand (15-30cm) | 3.2 |
| slope | Slope | 4.4 |
| whc_soil | Water holding capacity of soil | 2.52 |
| soil salinity | Soil salinity | 1.99 |
|  |  |  |
| ^*^VIF: Variance Inflation Factor | | |

# **Supplementary Figures**

| 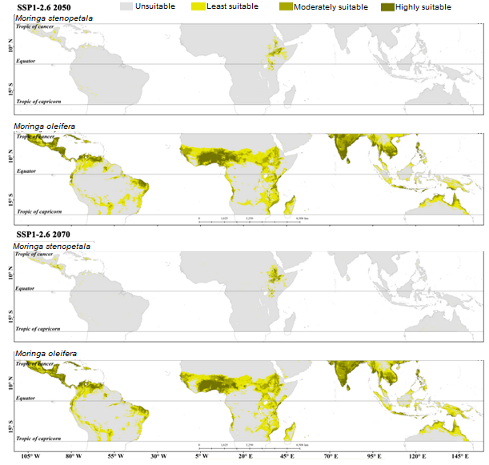 |
| --- |

Figure S1: Distribution of *Moringa oleifera* and *Moringa stenopetala* under SSP1-2.6 2050 (upper) and 2070 (lower). All maps were generated by authors of this work using ArcGIS 10.8.2 (https://www.arcgis.com/index.html).

| 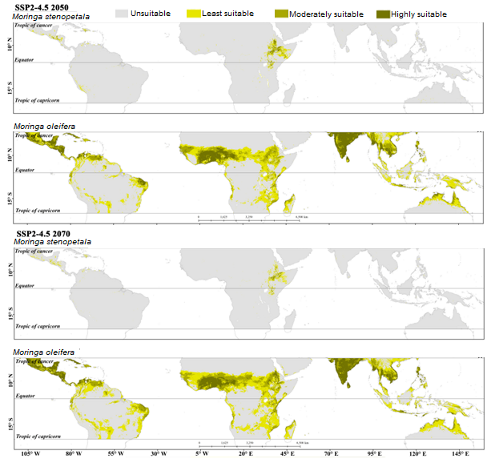 |
| --- |

Figure S2: Distribution of *Moringa oleifera* and *Moringa stenopetala* under SSP2-4.5 2050 (upper) and 2070 (lower). All maps were generated by authors of this work using ArcGIS 10.8.2 (https://www.arcgis.com/index.html).

| 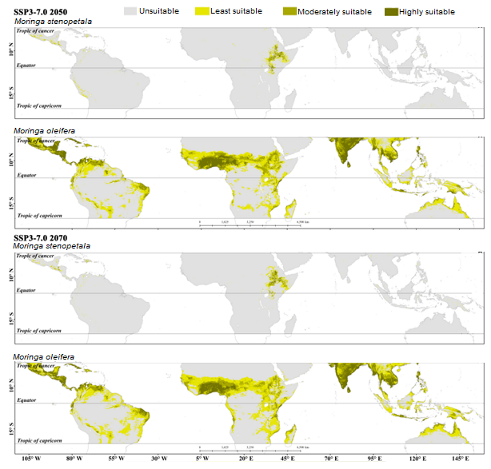 |
| --- |

Figure S3: Distribution of *Moringa oleifera* and *Moringa stenopetala* under SSP3-7.0 2050 (upper) and 2070 (lower). All maps were generated by authors of this work using ArcGIS 10.8.2 (https://www.arcgis.com/index.html).

| 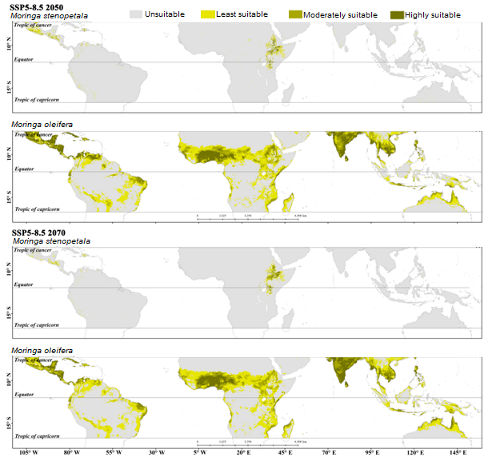 |
| --- |

Figure S4: Distribution of *Moringa oleifera* and *Moringa stenopetala* under SSP5-8.5 2050 (upper) and 2070 (lower). All maps were generated by authors of this work using ArcGIS 10.8.2 (https://www.arcgis.com/index.html).


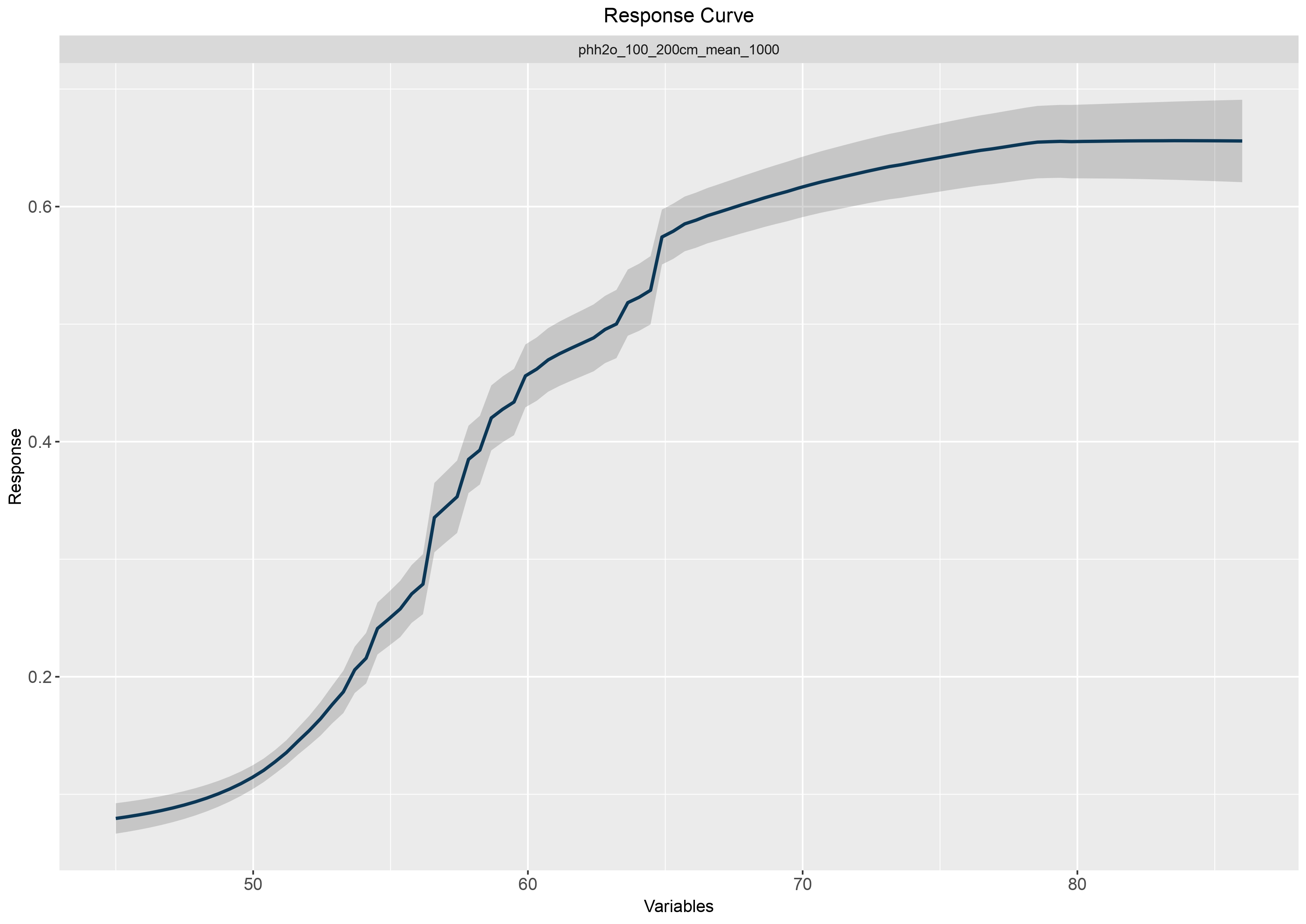


Figure S5: Variability curve of soil pH in distribution of *Moringa oleifera*


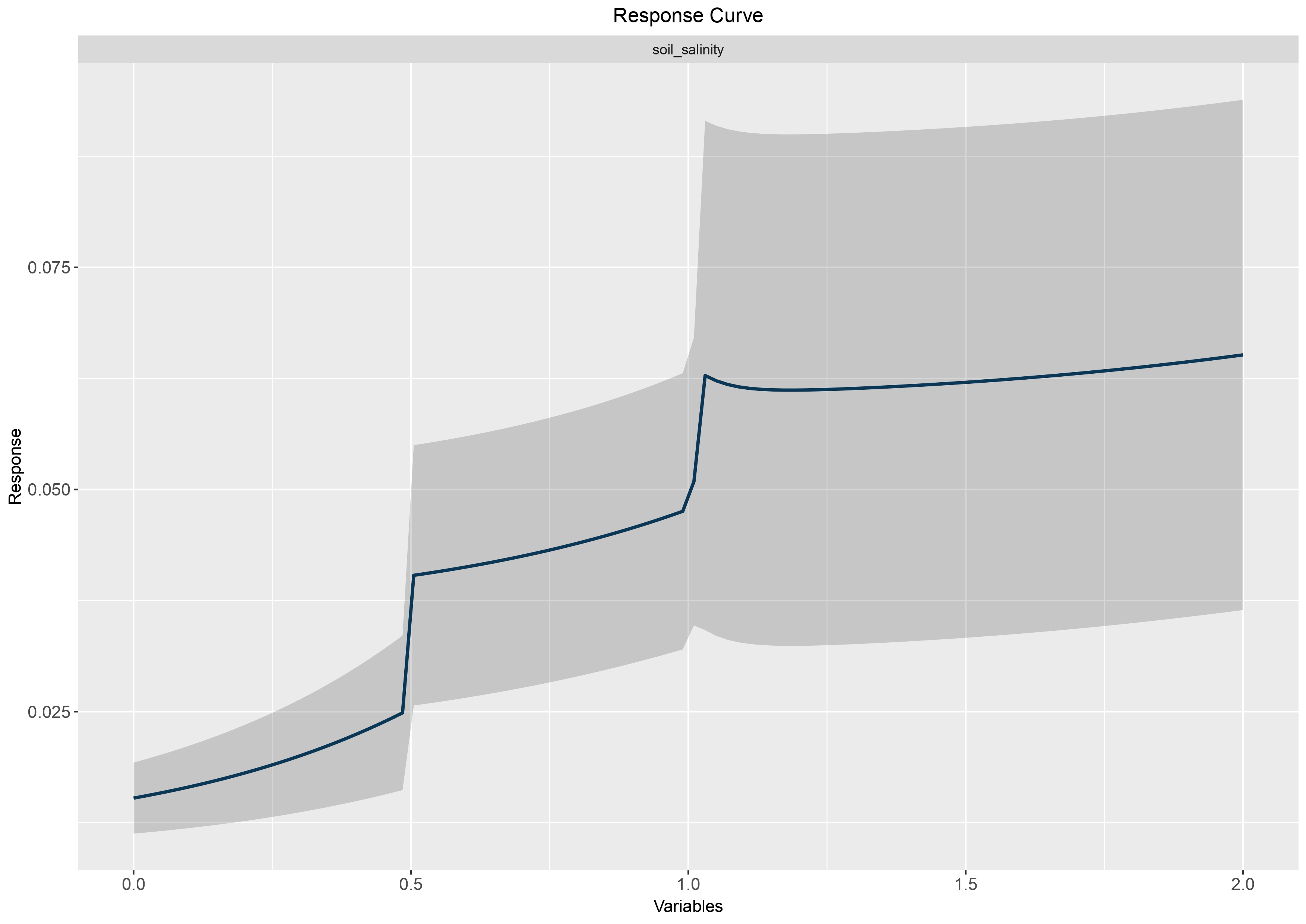


Figure S6: Variability curve of soil salinity in distribution of *Moringa stenopetala*
